# On Superfœtation

**Published:** 1810-04

**Authors:** 


### *To the Editors of the Medical and Physical Journal.*

Gentlemen,

THAT superfœtation was an attribute of all animal bodies was not at all questioned by our medical ancestors; and even at this comparatively enlightened period, the majority of medical practitioners have, by no means, made up their minds on the subject. This indecision has been kept up by occasional instances of twins being born in different states of growth. A case of this kind having happened to me within this month, and there having been but few such placed upon record within the last age, I have taken the liberty of sending it for insertion in your Journal, with a few remarks upon the doctrine of superfœtation in general.

A woman was delivered of her fourth child with the usual phænomena; but with the after-birth, there came away a fœtus seemingly of five month's growth, dead, and considerably flattened, but not at all putrid, although the skin had nearly the colour of lead. The only reason the woman could offer for such an occurrence, was, that when when five month's gone with child, she was so much frightened by a cow as to fall down. After that time, however, she never felt any particularly unpleasant sensation, although she had always been certain that she should have twins.

In every case of this kind that I have seen or heard of, the second fœtus has been much younger than it was in this instance. Here, however, we must allow, either that superfœtation had taken place, or that the woman had carried in her uterus a bulk of extraneous matter, which, reasoning a priori, we must have concluded, would inevitably have stimulated the uterus to contraction sufficient for its expulsion. The case, therefore, is interesting, inasmuch as it proves the great power which the living action going on in the uterus had, in enabling it to resist the stimulus produced by so large an extraneous body existing in its cavity.

There still, however, remain a few circumstances respecting superfœtation, which deserve notice.

No person who has attentively considered the anatomy of the gravid uterus, can for a moment suppose that superfœtation can happen after conception has taken place for some time, because it is impossible that the seminal fluid should get in contact with the ovarium; for not to mention the sides of the uterus being in perfect contact with the membranes within them, the semen could not by any possibility enter the cervix uteri, in consequence of its being stopped up by a gelatinous substance, so tough, that Dr. Hunter says, " upon attempting to pass a probe through it, the probe will as readily force its way through the hard substance of the uterus itself, as through the cementing jelly." We are not told, however, by any author, at what time this jelly is thrown out by the cervix, so that we do not know how long after conception the os uteri remains open.

Dr. Hamilton states, that "bitches from admitting a variety of dogs while they are in season, will bring forth puppies of these different species." This he explains, by the fact of the uterus of brutes being divided into cells or horns. Such an explanation, however, must be nugatory; because, although the uterus is divided into two or more cornua, there still is but one cervix uteri, which, as it is of course stopped up soon after conception takes place, would as certainly hinder the occurrence of a second conception, as in the human subject. Dr. Hamilton indeed, says, likewise, that " the ova in brutes do not attach themselves themselves to the uterus so early as in the human subject, but are supposed to receive their nourishment for some time by absorption.

This however, is an assertion most certainly without the smallest proof, and in my mind, without the smallest probability; nature never makes a variety in particular structures, unless it is to answer some end;—and why should not the ovum of an animal attach itself to that animal's uterus, as soon as the ovum of the human creature to the human uterus? There is, however, I believe, upon record, an instance of a black woman in America, bearing full-grown twins, one being a black child, and the other a mulatto. This case clearly puts Dr. Hamilton's distinctions between the human species and other animals out of the question. How, therefore, are these occurrences to be explained? Let us recur to the known facts attending conception; perhaps, we may then be led to conclusions concerning superfœtation, which have not, as yet, been published by any author on the subject.

Conception it would seem, is a work of time.—We know that the seminal fluid will take some time in passing along the Fallopian tubes to the ovarium; it is well known also, that the ovum is a considerable time in passing backwards into the uterus. The precise time, indeed, is unknown, but in bitches it must be considerable, perhaps some days, because we know, that before the ovum enters the uterus, there is that peculiar fluid or coagulable matter thrown out by its internal surface, which, as the fœtus descends, covered by its chorion and amnion, forms the decidua vera and decidua reflexa.

This gelatinous flaky matter will prevent the application of fresh semen to the remaining ovarium, as certainly as the jelly stopping up the cervix uteri, which probably, indeed, is thrown out at the same time, therefore, *after* this time, a fresh conception is impossible. We must, however, conclude, that *before* this matter is thrown out, seminal fluid in a second connexion, may find its way into the remaining horn of the bitch's uterus, or (by parity of reasoning) into the remaining Fallopian tube of the human uterus; and that, therefore, we may strictly say, that superfœtation *can* take place in the very early stages of pregnancy, although not after the ovum is fairly lodged in the cavity of the uterus.

H.

*February* 24, 1810.

